# Impact pathways of personality and psychosocial stress on depression among adult community residents in China: a fuzzy-set qualitative comparative analysis

**DOI:** 10.3389/fpsyg.2024.1375698

**Published:** 2024-07-22

**Authors:** Xi Yang, Liuruyu Yu, Shengming Zhang, Zhaoguo Wei, Gaoqiang Xie, Jianhong Wang

**Affiliations:** ^1^Shenzhen Mental Health Center, Shenzhen Kangning Hospital, Shenzhen, Guangdong, China; ^2^Clinical Research Institute, Institute of Advanced Clinical Medicine, Peking University, Beijing, China; ^3^Affiliated Mental Health Center, Southern University of Science and Technology, Shenzhen, Guangdong, China; ^4^Key Laboratory of Epidemiology of Major Diseases (Peking University), Ministry of Education, Beijing, China

**Keywords:** depression, personality traits, life events, psychological stress, fs-QCA

## Abstract

**Objective:**

Depression is a common mental illness with a high prevalence rate and is a significant contributor to the global burden of diseases. Various factors are associated with depression, and its etiology is complex. Instead of focusing on single-factor effects, this study aimed to explore a combination of high-risk factor sets for depression among adult community residents.

**Methods:**

We conducted a cross-sectional survey in Shenzhen, China, from January 2021 to March 2021. A simple sampling method was used to enroll participants. A total of 1,965 adult residents completed the survey and were assessed using the Patient Health Questionnaire-9 (PHQ-9), the Eysenck Personality Questionnaire-Revised Short Scale for Chinese (EPQ-RSC), and the Psychosocial Stress Survey for Groups (PSSG). The fuzzy-set qualitative comparative analysis method was used to explore the high-risk factor sets for depression among adult community residents.

**Results:**

The prevalence of depression among the surveyed adult residents in Shenzhen was 6.36%. The mean scores of PHQ-9 were higher among women and unmarried residents. The combination of low extroversion (e) and high neuroticism (N) in personality traits, along with high scores for life events (V), negative emotional responses to events (G), positive emotional responses to events (O), and positive coping styles to events (I) (denoted as e^*^N^*^V^*^G^*^O^*^I) constituted a high-risk factor set for depression. The overall consistency was 0.843, and the overall coverage was 0.330.

**Conclusion:**

Our study suggested that stressful life events together with personality traits including neuroticism and introversion serve as crucial factors for depression among adult community residents, regardless of the coping strategies they adopt. This study provides data for developing comprehensive interventions such as regulating neuroticism and introversion levels and reducing stressors to prevent the occurrence of depression among adult community residents.

## 1 Introduction

Depression is a major public mental health problem worldwide, which impacts life with a high incidence and is among the greatest contributors to the global burden of diseases, including physical health and risk behaviors (Prince et al., 2007; James et al., 2018). According to the China Mental Health Survey, the lifetime prevalence and 12-month prevalence of depressive disorders in China were 6.8 and 3.6%, respectively (Huang et al., 2019). Since 2010, depressive disorders have been the second leading cause of years lived with disability, and people with depressive disorders have reported significant social impairment in China (Yang et al., 2013; Lu et al., 2021). Numerous factors are associated with depression, and the etiology is complex. The diathesis–stress theory of depression, proposed three decades ago by Monroe and Simons (1991), suggests that stress activates vulnerability, transforming a potential predisposition into psychopathology. The theory highlights the synergistic relationship between diathesis and stress.

Personality traits have been identified as diathesis factors for depression—high scores of neuroticism increase the risk of depression (Feizi et al., 2014; Nouri et al., 2019; Sheldon et al., 2021), whereas high extroversion has been considered a protective factor against stress response in mood disorders (Liu et al., 2022; Nin et al., 2023). López et al. (2017) found that the primary and overall personality dimensions explained 28% of the variance in depression. Studies indicated that vulnerable personality traits lead to the onset and development of depression through multiple pathways (Klein et al., 2011). In addition, another perspective suggested that the differences in depression cannot solely be attributed to personality traits (Milić et al., 2019).

**Figure 1:**
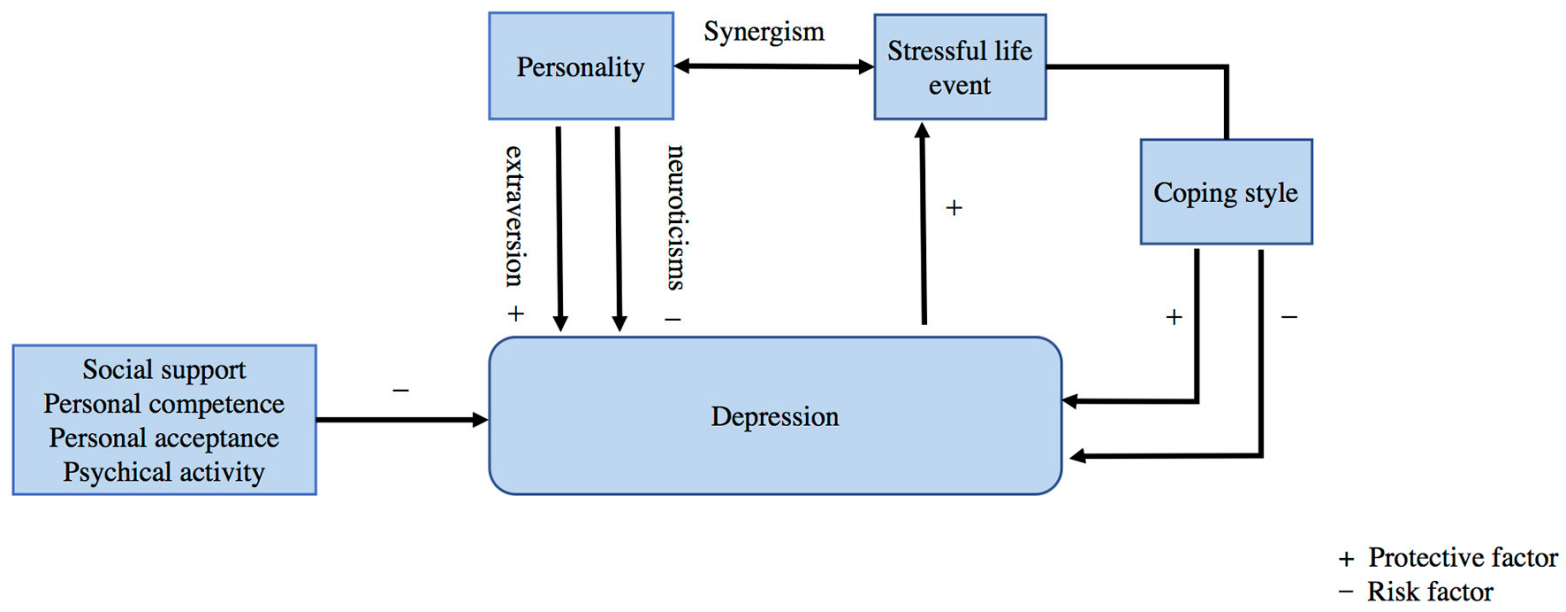
Diathesis-stress theory model of depression.

According to the diathesis–stress theory, stress arises from life events that disrupt the psychological balance. Stress can catalyze the development of a disorder, and stressful life events (SLEs) are a predictor of susceptibility to subsequent depression (Tennant, 2002; Ngasa et al., 2017; Bjørndal et al., 2023; Crouse et al., 2024). The transactional theory of stress and coping indicates that individuals assess situations and adopt coping strategies, typically categorized as problem-focused, emotion-focused, or avoidant. Many studies suggested that coping strategies during stress mediate depressive symptoms. Active coping is associated with decreased depression levels, whereas frequent use of maladaptive strategies is related to increased depressive symptoms (Stikkelbroek et al., 2016; Kugbey et al., 2018; Pelekanakis et al., 2022; Alshowkan et al., 2023; Kumar et al., 2023). A dynamic precursor model suggested that depression and stress are mutually predictive factors. SLEs predict subsequent neuroticism at three out of four intervals (Goldstein et al., 2020). However, a recent study presented a different perspective, showing that the level of depression may be relatively high even if a moderate level of adaptability and active coping strategies are applied during stressful life events (Huang et al., 2022). Further discussion on the mutual relationship between SLEs, coping strategies, and depression remains urgently needed. Moreover, other factors related to personality traits and community networks, such as social support and utilization, physical activity, personal competence, and acceptance, are considered potential protective factors against depression (Choi et al., 2022; Ferber et al., 2022; Lu et al., 2023; Iovoli et al., 2024). The diathesis–stress theory of depression is illustrated in Figure 1.

Previous studies have focused on the individual effects of factors such as personality traits, life events, and social support. However, several other risk factors may exist simultaneously in actual situations. The combination of various features is complex and variable for individuals. In this study, we aimed to identify the different combinations of personality traits, SLEs, and coping strategies that have greater potential to cause depression among adult community residents.

The fuzzy-set qualitative comparative analysis (fs-QCA) is a research method that blends qualitative and quantitative approaches, combining fuzzy set theory and logic principles with QCA as proposed by Ragin (2000). fs-QCA has been widely used in public health studies in recent years (Breuer et al., 2018), including evaluations of public health interventions and discussions of the risk factors of occupational stress (Quan et al., 2022). In this study, we applied fs-QCA to analyze the effects of personality traits, SLEs, and coping styles during stress among adult community residents in Shenzhen, China and to identify high-risk factor sets for depression. This study aimed to provide a theoretical basis for effectively coping with depression and planning intervention measures.

## 2 Methods

### 2.1 Study population

In this study, we used a simple sampling method to enroll community residents in Shenzhen, China, from January 2021 to March 2021. The inclusion criteria were as follows: (1) individuals aged 18 and above, (2) those who have been a resident of a community living center in Shenzhen for over 6 months, and (3) those who provided informed consent. The exclusion criteria were as follows: (1) individuals who experienced difficulties in understanding the questionnaires and (2) those who were unable to complete the investigation for physical or psychological reasons.

#### 2.1.1 Sample size

The data were derived from the Mental Health Study in Shenzhen (MHSS). The sample size of the MHSS was estimated using Power Analysis and Sample Size (PASS) software. Based on the preliminary data, the prevalence rate of depression was calculated as 0.04 (Huang et al., 2019), Cronbach's α as 0.05, and the permissible error as 0.01. Considering a 20% drop-out rate, the number of samples as calculated by PASS software was 1,770. According to previous literature, a sample size of 15–20 was considered suitable for the application of QCA. However, published examples of analyses recommend an extension of QCA to a sample size >50 (Farrugia, 2019).

#### 2.1.2 Sampling process

**Figure 2:**
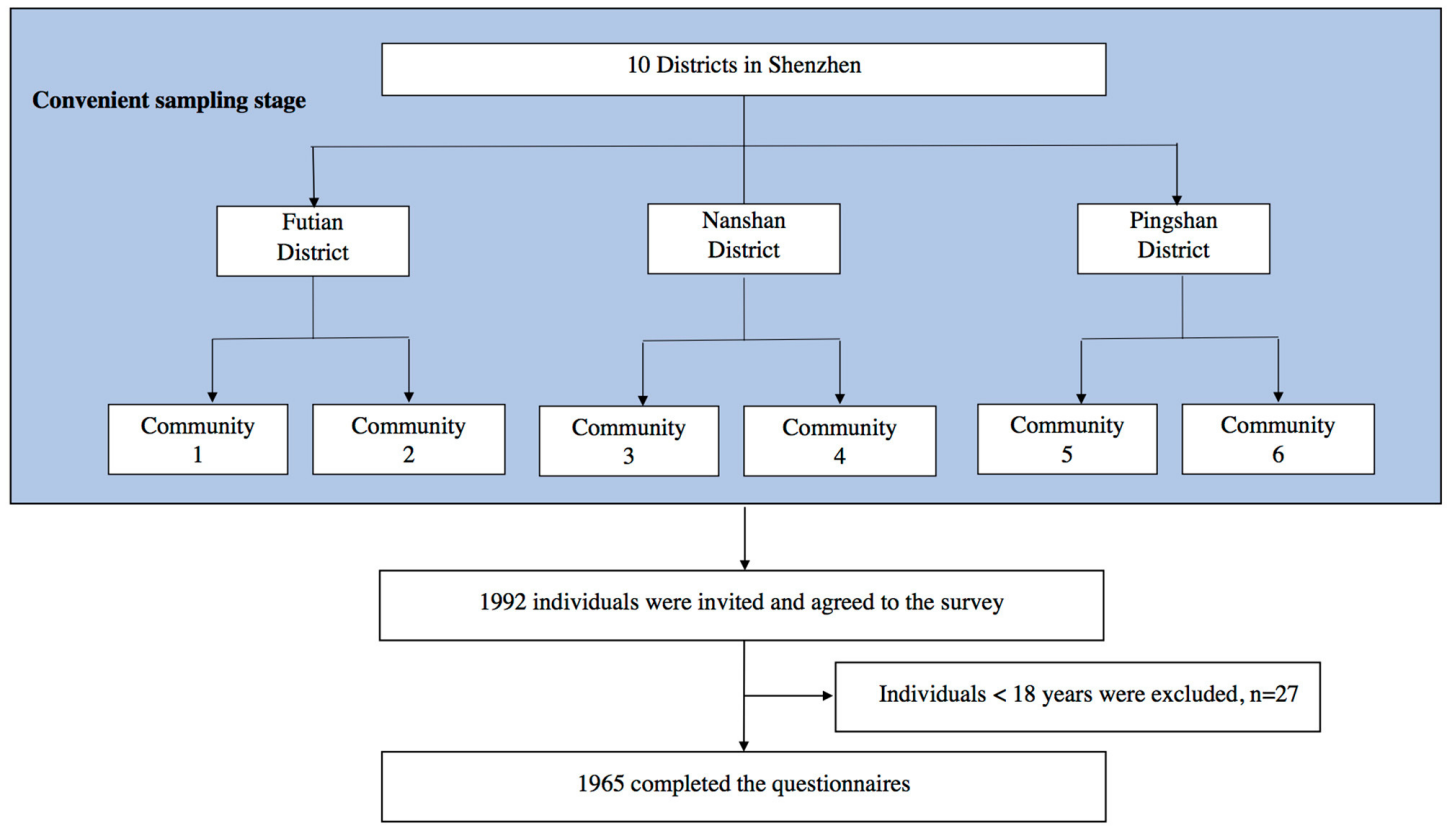
The sampling roadmap of the study.

First, we conveniently selected three districts, namely, Futian, Nanshan, and Pingshan, from the ten districts of Shenzhen. Second, we selected two communities in each district, making a total of six communities. Finally, we recruited volunteers to participate in the survey by posting posters and sending WeChat messages in the selected communities. A total of 1,992 community residents were invited to participate of whom 1,965 completed the questionnaire and were finally included. The sampling roadmap is illustrated in Figure 2.

#### 2.1.3 Investigation process

Before the investigation, the research team recruited investigators with experience in the primary mental health field and organized training courses for them. The training content included standardized evaluation procedures and data management measures. All participants completed the survey individually under the face-to-face guidance of the investigators. This study was approved by the Ethics Committee of Shenzhen Kangning Hospital.

### 2.2 Measurements

A self-administered questionnaire and three standardized scales were used to collect data, including sociodemographic characteristics, the level of depression, personality, and psychosocial stress.

#### 2.2.1 Sociodemographic characteristics

Characteristics such as age, sex, body mass index (BMI), education, occupation, marriage status, individual income, family income, chronic disease, number of chronic diseases, smoking, and alcohol use were included.

#### 2.2.2 Patient Health Questionnaire-9

The Patient Health Questionnaire-9 (PHQ-9) is a self-administered scale used to screen, define, and quantify the severity of depressive symptoms over the past 2 weeks. It has a high sensitivity comparable to semi-structured interviews (Kroenke, 2012). The Chinese version of PHQ-9 was validated among the general Chinese population, indicating an internal consistency reliability with a Cronbach's alpha of 0.86. Hence, the Chinese version of the PHQ-9 is a valid and efficient tool for screening depression among individuals in the community (Wang et al., 2014). A cut-off score of 10 or above was adopted to screen for depression, which maximized combined overall sensitivity and specificity and those for subgroups (Levis et al., 2019).

#### 2.2.3 Eysenck Personality Questionnaire-Revised Short Scale for Chinese

The Eysenck Personality Questionnaire-Revised Short Scale (EPQ-RS) is a self-report scale developed by Eysenck et al. (1985) and Eysenck (1996) to measure personality traits. The dimensions of the scale included extroversion, neuroticism, psychoticism, and lying. A high score in the extraversion dimension indicates extroversion (E) and a low score indicates introversion (e). In the neuroticism dimension, a high score indicates a stronger emotional reaction (N) and a low score indicates a weaker emotional reaction (n). A high score in psychoticism indicates loneliness, difficulty adapting to the external environment, and a tendency to provoke others (P), whereas a low score indicates normal (p). The Eysenck personality Questionnaire-revised Short Scale for Chinese (EPQ-RSC) has high reliability and validity (Qian et al., 2000).

#### 2.2.4 Questionnaire of psychosocial stress survey for groups

A questionnaire of the Psychosocial Stress Survey for Groups (PSSG) was compiled by Jiang in Chinese. It consists of 44 items, reflecting three levels of psychological stress theory: life events (L), emotional responses to events (E), and coping styles to events and emotional responses (C). The emotional response to events includes positive emotional experience (PE) and negative emotional experience (NE). The coping styles also include positive coping (PC) and negative coping (NC). The retest reliability values of the PSSG total score, L, NE, PE, NC, and PC were 0.88, 0.70, 0.83, 0.76, 0.80, and 0.81, respectively (Jiang, 1998).

### 2.3 Statistical analysis

All analyses were performed with STATA16.0 and SAS 9.4. An analysis of variance (ANOVA) was used to compare the PHQ-9 scores of sex, age, marital status, education level, income, alcohol drinking, and smoking using subgroups. After adjusting for age, sex, education, marital status, alcohol drinking, and smoking, a partial correlation analysis was conducted to analyze the correlation between the PHQ-9 scores and personality traits and psychosocial stress. Subsequently, fs-QCA was performed according to Quan et al. (2022) to test different combinations and dimensionality-reduced factor sets among the variables. This study used eight variables for fs-QCA: extroversion (E/e), neuroticism (N/n), psychoticism (P/p), life events (V/v), negative emotional responses to events (G/g), positive emotional responses to events (O/o), negative coping styles to events (A/a), and positive coping styles to events (I/i).

## 3 Results

### 3.1 Prevalence of depression among adult community residents

Among the 1,965 participants, the prevalence of depression was 6.36%. Table 1 presents the relationships between PHQ-9 scores and sociodemographic factors. The mean scores of PHQ-9 were higher among women and unmarried residents. Significant differences in PHQ-9 scores were observed by age group, education level, and alcohol use status. No significant differences were found in the depression levels among residents of different economic levels and smoking status.

**Table 1:** Participants' baseline characteristics.

**Characteristic**	***N***	**PHQ-9**
**Sex**		
Men	783	3.24 ± 3.48
Women	1,182	3.67 ± 3.81
*P* ^*^		0.0121
**Age**		
≤ 34	990	3.89 ± 3.66
35–44	598	3.38 ± 3.82
≥45	377	2.67 ± 3.41
*P* ^*^		0.0000
**Marital status**		
Single	666	4.27 ± 3.78
Others	1,299	3.11 ± 3.58
*P* ^*^		0.0000
**Education**		
Primary education or less	42	3.31 ± 3.88
Secondary education	654	2.97 ± 3.54
College education or above	1,269	3.78 ± 3.73
*P* ^*^		0.0000
**Individual income/month, CNY**		
0–3,000	269	3.81 ± 3.89
3,000–4,999	624	3.41 ± 3.70
5,000–7,999	688	3.47 ± 3.62
≥8,000	384	3.48 ± 3.66
*P* ^*^		0.5172
**Family income/month, CNY**		
0–6,000	249	3.38 ± 3.89
6,000–9,999	500	3.16 ± 3.36
10,000–19,999	709	3.75 ± 3.80
≥20,000	507	3.54 ± 3.73
*P* ^*^		0.0523
**Alcohol**		
No	1,481	3.35 ± 3.59
Ever	154	3.33 ± 3.32
Current	330	4.25 ± 4.17
*P* ^*^		0.0003
**Smoking**		
No	1,635	3.51 ± 3.66
Ever	75	3.6 ± 4.50
Current	255	3.38 ± 3.61
*P* ^*^		0.8340

PHQ-9, The Patient Health Questionnaire-9.

One-way ANOVA was used to compare the difference in the mean score of PHQ between groups. ^*^Indicates *P* < 0.05.

### 3.2 Relationship between personality, psychosocial stress, and depression

Table 2 shows the results of the partial correlation analysis among the included factors after adjusting for age, sex, education, marital status, alcohol use, and smoking. Statistically significant correlations were observed between all three personality traits, certain psychological stress dimensions (i.e., life events, NE, PE, and PC), and depression among adult community residents (*P* < 0.05). The correlation coefficients between neuroticism and depression were >0.5.

**Table 2:** Pearson correlation coefficient between depression and personality, psychosocial stress.

**Variables**	**PHQ-9**
**EPQ-RSC**	
Exotropism (E)	−0.1903^a^
Neuroticism (N)	0.5312^a^
Psychoticism (*P*)	0.0449^a^
**PSSG**	
Life events (L)	0.3550^a^
Negative emotional responses to events (NE)	0.3020^a^
Positive emotional responses to events (PE)	0.2224^a^
Negative Coping styles to events and emotional (NC)	0.0395
Positive Coping styles to events and emotional (PC)	0.4186^a^

PHQ-9, The Patient Health Questionnaire-9; ESQ-RSC, Eysenck personality Questionnaire-revised Short Scale for Chinese; PSSG, Questionnaires of psychosocial stress survey for groups. Adjusted for age, sex, education, marital status, drink alcohol and smoking.

^a^Indicates *P* < 0.05.

### 3.3 High-risk factor sets of mental health

To explore the high-risk factor sets of depression among adult community residents in Shenzhen, we conducted the fs-QCA, including personality traits and psychosocial stress. The set-gen command was used to fuzzify the data to a range between 0 and 1 without varying the original data distribution. The independent variables were extroversion (E/e), neuroticism (N/n), psychoticism (P/p), life events (V/v), negative emotional responses to events (G/g), positive emotional responses to events (O/o), negative coping styles to events (A/a), and positive coping styles to events (I/i). The dependent variable was the PHQ-9 score.

To configure the complexity of causality, we constructed a truth table to systematically analyze all the logically possible combinations of conditional variables (Supplementary material 1). The consistency test results revealed 247 common sets with statistical significance (*P* < 0.05) in depression. Dimensionality reduction was performed using the reduce command (Table 3). The results showed that the high-risk factor set for depression was low extroversion (e) and high neuroticism (N) combined with high life events (V), high negative emotional responses to events (G), high positive emotional responses to events (O), and high positive coping styles to events (I) (denoted as e^*^N^*^V^*^G^*^O^*^I). According to Ragin (2008), the consistency coefficient should be more than 0.75, and the coverage rate should be more than 0.25. Our model configuration has a consistency of 0.843 (>0.75) and an overall coverage of 0.330, suggesting that it sufficiently explains the path of depression for 30% of the cases.

**Table 3:** Dimensionality reduction for common sets of PHQ-9.

**Set**	**Raw coverage**	**Unique coverage**	**Solution consistency**	**Num bestfit**
**PHQ-9**				
e^*^N^*^V^*^G^*^O^*^I	0.330	0.330	0.843	165
Overall consistency	0.843			
Overall coverage	0.330			

PHQ-9, The Patient Health Questionnaire-9; ESQ-RSC, Eysenck personality Questionnaire-revised Short Scale for Chinese; PSSG, Questionnaires of psychosocial stress survey for groups.

^*^Use the reduce command to obtain the final factor combination set.

## 4 Discussion

From the sociological perspective, individuals exhibit a complex combination of characteristics. This study examined the combined relationship between depression, personality traits, and psychosocial stress using fs-QCA. We identified that the combination of low extroversion (e and high neuroticism (N) in personality traits with high life events (V), high negative emotional responses to events (G), high positive emotional responses to events (O), and high positive coping styles to events (I) constitutes a high-risk factor set for depression. However, this factor set represents sufficient but not the necessary conditions for depression, indicating that individuals with a combination of these factors are more likely to experience depression compared to others.

Many studies have discussed the relationship between personality traits and depression, and personality has been considered a key factor in depression. High-order personality factors, including neuroticism and extroversion, are significantly and consistently associated with depression (Grav et al., 2012). A study on a community-based lifespan sample in the United States demonstrated that high neuroticism and low extroversion were the most significant predictors of depression in a machine-learning model (Yang et al., 2024). Neuroticism, a partially heritable personality trait representing high emotionality and sensitivity (Matthews and Whiteman, 2009), maybe the strongest predictor of depression (Enns and Cox, 1997; Nouri et al., 2019; Crouse et al., 2024). Previous studies on personality traits and depression have overlooked the correlation between introversion and depression. However, research suggests a promising association between introversion and the outcomes of depression, with introversion potentially representing a heritable trait of etiologic significance (Janowsky, 2001). Introverts tend to report fewer pleasant experiences (DeMeo et al., 2023). Our analysis supports the idea that a combination of high neuroticism and low extroversion in personality dimensionality is a high-risk factor set for depression.

SLEs are consistently considered having significant association with depressive symptoms (Kessler, 1997; Kendler et al., 1999; Hammen, 2005; Stroud et al., 2008). A longitudinal study suggested a direct correlation between increased neuroticism, aggregation of SLEs, and more severe depressive symptoms. It demonstrated that negative life events partially mediate the relationship between neuroticism and depression (Zheng et al., 2024). Maladaptive coping strategies are associated with more depressive symptoms, whereas resilience and positive coping styles moderate the relationship between SLEs and depression (Stikkelbroek et al., 2016; Zhao et al., 2021; Huang et al., 2022). However, some studies suggested that, despite significant heterogeneity in how people respond to stress, SLEs and perceived stress are central risk factors in the complex depression network (Tennant, 2002; Ngasa et al., 2017; Goldstein et al., 2020; Bjørndal et al., 2023; Iovoli et al., 2024). Our study found that a high score in life events, combined with both positive and negative emotional responses and positive coping styles, constitute a high-risk factor set for depression. This finding is consistent with the result of a recent study showing that the level of depression is relatively high even if a moderate level of adaptability and positive coping strategies are adopted in stressful life environments (Huang et al., 2022). We suggest that stress plays an independent and significant role in depression, with stressors, along with personality predispositions, forming a risk set for depression.

This study has certain limitations. First, the fs-QCA method used is a qualitative analysis and does not explain the quantitative effect of variables. Second, due to the cross-sectional approach used in our study, the association between depression and personality and stress cannot be interpreted causally. Third, volunteer bias may occur due to the sampling process. The prevalence of depression in our study was 6.36%, higher than the 12-month prevalence of depressive disorders in the China Mental Health Survey (Huang et al., 2019), possibly indicating that individuals with depression were more likely to participate. Although their participation may increase the statistical power, it may not impact the magnitude of the observed associations.

## 5 Conclusion

Rather than limiting to single-factor effects, our study explored the combined high-risk factor sets of personality traits and psychological stress on depression in adult community residents using the fs-QCA method. The findings of the present study suggest that SLEs combined with personality traits, including neuroticism and introversion, serve as crucial factors in depression among adult community residents, regardless of whether they use positive or negative coping strategies. To reduce the prevalence of depression among adult community residents, comprehensive approaches that regulate the levels of neuroticism and introversion and actively integrate social resources to mitigate stressors should be adopted.

## Data availability statement

The raw data supporting the conclusions of this article will be made available by the authors, without undue reservation.

## Ethics statement

The studies involving humans were approved by Ethics Committee of Shenzhen Kangning Hospital. The studies were conducted in accordance with the local legislation and institutional requirements. The participants provided their written informed consent to participate in this study.

## Author contributions

XY: Writing – original draft, Methodology. LY: Methodology, Writing – original draft. SZ: Project administration, Writing – review & editing. ZW: Project administration, Supervision, Writing – review & editing. GX: Methodology, Supervision, Writing – review & editing. JW: Funding acquisition, Resources, Supervision, Writing – review & editing.
