# Supplementary material for: Impact pathways of personality and psychosocial stress on depression among adult community residents in China: a fuzzy-set qualitative comparative analysis
Source: Front Psychol. 2024 Jul 22;15:1375698. doi: 10.3389/fpsyg.2024.1375698 (PMC11302050; doi:10.3389/fpsyg.2024.1375698)
Supplement: Supplementary file 1 [file Table_1.docx]

Supplement

Table Consistency test for common sets of PHQ-9

| **Set** | **YCons** | **N-consistency** | | |  | **Set value**  **（0.700）** | | **Num bestfit** |
| --- | --- | --- | --- | --- | --- | --- | --- | --- |
|  |  | **NCons** | **F** | **P** |  | **F** | **P** |  |
| EPQ-RSC & PSSG |  |  |  |  |  |  |  |  |
| penvgoai | 0.627 | 0.946 | 337.8 | 0.000 |  | 24.91 | 0.000 | 38 |
| penvgoaI | 0.703 | 0.945 | 188.38 | 0.000 |  | 0.05 | 0.815 | 13 |
| penvgoAi | 0.65 | 0.957 | 339.97 | 0.000 |  | 11.73 | 0.001 | 20 |
| penvgoAI | 0.705 | 0.95 | 200.61 | 0.000 |  | 0.1 | 0.749 | 5 |
| penvgOai | 0.684 | 0.96 | 314.14 | 0.000 |  | 1.36 | 0.244 | 9 |
| penvgOaI | 0.741 | 0.951 | 172.23 | 0.000 |  | 8.52 | 0.004 | 8 |
| penvgOAi | 0.691 | 0.958 | 289.36 | 0.000 |  | 0.39 | 0.532 | 13 |
| penvgOAI | 0.741 | 0.95 | 167.18 | 0.000 |  | 8.28 | 0.004 | 7 |
| penvGoai | 0.677 | 0.958 | 274.71 | 0.000 |  | 2.44 | 0.118 | 3 |
| penvGoaI | 0.725 | 0.952 | 172.35 | 0.000 |  | 2.72 | 0.099 | 2 |
| penvGoAi | 0.686 | 0.957 | 261.37 | 0.000 |  | 0.96 | 0.327 | 0 |
| penvGoAI | 0.724 | 0.952 | 173.97 | 0.000 |  | 2.58 | 0.108 | 1 |
| penvGOai | 0.699 | 0.954 | 271.64 | 0.000 |  | 0.01 | 0.943 | 6 |
| penvGOaI | 0.746 | 0.941 | 143.68 | 0.000 |  | 11.27 | 0.001 | 9 |
| penvGOAi | 0.708 | 0.95 | 229.84 | 0.000 |  | 0.36 | 0.548 | 10 |
| penvGOAI | 0.748 | 0.934 | 125.87 | 0.000 |  | 12.24 | 0.000 | 12 |
| penVgoai | 0.703 | 0.941 | 181.75 | 0.000 |  | 0.03 | 0.856 | 13 |
| penVgoaI | 0.747 | 0.933 | 106.96 | 0.000 |  | 10.51 | 0.001 | 2 |
| penVgoAi | 0.711 | 0.943 | 172.81 | 0.000 |  | 0.57 | 0.452 | 7 |
| penVgoAI | 0.745 | 0.934 | 111.98 | 0.000 |  | 9.12 | 0.003 | 9 |
| penVgOai | 0.742 | 0.95 | 171.77 | 0.000 |  | 9.25 | 0.002 | 3 |
| penVgOaI | 0.777 | 0.938 | 100.57 | 0.000 |  | 33.73 | 0.000 | 1 |
| penVgOAi | 0.744 | 0.946 | 158.68 | 0.000 |  | 10.28 | 0.001 | 3 |
| penVgOAI | 0.774 | 0.933 | 93.71 | 0.000 |  | 30.67 | 0.000 | 6 |
| penVGoai | 0.729 | 0.945 | 147.08 | 0.000 |  | 3.92 | 0.048 | 1 |
| penVGoaI | 0.764 | 0.936 | 94.76 | 0.000 |  | 19.13 | 0.000 | 2 |
| penVGoAi | 0.729 | 0.947 | 154.31 | 0.000 |  | 3.86 | 0.050 | 1 |
| penVGoAI | 0.759 | 0.936 | 98.59 | 0.000 |  | 16.01 | 0.000 | 0 |
| penVGOai | 0.75 | 0.936 | 126.13 | 0.000 |  | 13.77 | 0.000 | 10 |
| penVGOaI | 0.785 | 0.917 | 62.15 | 0.000 |  | 42.18 | 0.000 | 9 |
| penVGOAi | 0.75 | 0.933 | 121.55 | 0.000 |  | 13.71 | 0.000 | 12 |
| penVGOAI | 0.78 | 0.907 | 55.19 | 0.000 |  | 37.11 | 0.000 | 16 |
| peNvgoai | 0.717 | 0.93 | 127.53 | 0.000 |  | 1.19 | 0.276 | 9 |
| peNvgoaI | 0.765 | 0.909 | 56.82 | 0.000 |  | 19.63 | 0.000 | 9 |
| peNvgoAi | 0.724 | 0.932 | 126.14 | 0.000 |  | 2.38 | 0.123 | 8 |
| peNvgoAI | 0.762 | 0.912 | 57.65 | 0.000 |  | 16.77 | 0.000 | 11 |
| peNvgOai | 0.759 | 0.942 | 125.45 | 0.000 |  | 18.24 | 0.000 | 3 |
| peNvgOaI | 0.799 | 0.91 | 40.78 | 0.000 |  | 55.97 | 0.000 | 4 |
| peNvgOAi | 0.76 | 0.937 | 110.61 | 0.000 |  | 18.03 | 0.000 | 2 |
| peNvgOAI | 0.792 | 0.915 | 50.39 | 0.000 |  | 46.37 | 0.000 | 8 |
| peNvGoai | 0.753 | 0.939 | 111.31 | 0.000 |  | 12.64 | 0.000 | 0 |
| peNvGoaI | 0.787 | 0.909 | 42.08 | 0.000 |  | 38.27 | 0.000 | 3 |
| peNvGoAi | 0.753 | 0.936 | 103.32 | 0.000 |  | 12.72 | 0.000 | 0 |
| peNvGoAI | 0.784 | 0.914 | 47.43 | 0.000 |  | 33.08 | 0.000 | 2 |
| peNvGOai | 0.771 | 0.927 | 87.99 | 0.000 |  | 27.99 | 0.000 | 6 |
| peNvGOaI | 0.806 | 0.87 | 11.46 | 0.001 |  | 70.57 | 0.000 | 20 |
| peNvGOAi | 0.777 | 0.921 | 72.53 | 0.000 |  | 32.21 | 0.000 | 3 |
| peNvGOAI | 0.8 | 0.875 | 15.62 | 0.000 |  | 56.92 | 0.000 | 25 |
| peNVgoai | 0.775 | 0.91 | 50.46 | 0.000 |  | 25.81 | 0.000 | 4 |
| peNVgoaI | 0.809 | 0.874 | 10.99 | 0.001 |  | 61.48 | 0.000 | 10 |
| peNVgoAi | 0.774 | 0.916 | 58.59 | 0.000 |  | 25.86 | 0.000 | 1 |
| peNVgoAI | 0.809 | 0.88 | 13.55 | 0.000 |  | 64.43 | 0.000 | 11 |
| peNVgOai | 0.806 | 0.922 | 49.95 | 0.000 |  | 67.14 | 0.000 | 0 |
| peNVgOaI | 0.833 | 0.879 | 7.01 | 0.008 |  | 124.13 | 0.000 | 4 |
| peNVgOAi | 0.802 | 0.92 | 48.58 | 0.000 |  | 61.02 | 0.000 | 2 |
| peNVgOAI | 0.831 | 0.882 | 8.87 | 0.003 |  | 119.56 | 0.000 | 6 |
| peNVGoai | 0.793 | 0.917 | 45.97 | 0.000 |  | 42.13 | 0.000 | 0 |
| peNVGoaI | 0.828 | 0.86 | 2.44 | 0.118 |  | 99.81 | 0.000 | 8 |
| peNVGoAi | 0.788 | 0.918 | 48.68 | 0.000 |  | 37.57 | 0.000 | 0 |
| peNVGoAI | 0.823 | 0.866 | 4.69 | 0.030 |  | 91.27 | 0.000 | 10 |
| peNVGOai | 0.806 | 0.899 | 27.54 | 0.000 |  | 64.51 | 0.000 | 5 |
| peNVGOaI | 0.846 | 0.797 | 5.98 | 0.015 |  | 169.61 | 0.000 | 49 |
| peNVGOAi | 0.81 | 0.893 | 22.16 | 0.000 |  | 73.85 | 0.000 | 9 |
| peNVGOAI | 0.845 | 0.794 | 7.33 | 0.007 |  | 183.47 | 0.000 | 56 |
| pEnvgoai | 0.583 | 0.954 | 509.4 | 0.000 |  | 68.32 | 0.000 | 60 |
| pEnvgoaI | 0.675 | 0.953 | 273.01 | 0.000 |  | 2.94 | 0.086 | 11 |
| pEnvgoAi | 0.593 | 0.961 | 517.19 | 0.000 |  | 55.23 | 0.000 | 70 |
| pEnvgoAI | 0.664 | 0.955 | 295.39 | 0.000 |  | 5.81 | 0.016 | 23 |
| pEnvgOai | 0.65 | 0.965 | 431.72 | 0.000 |  | 13.37 | 0.000 | 19 |
| pEnvgOaI | 0.719 | 0.954 | 227.97 | 0.000 |  | 2.02 | 0.155 | 3 |
| pEnvgOAi | 0.655 | 0.961 | 394.24 | 0.000 |  | 10.68 | 0.001 | 21 |
| pEnvgOAI | 0.712 | 0.953 | 233.48 | 0.000 |  | 0.74 | 0.388 | 10 |
| pEnvGoai | 0.651 | 0.964 | 388.37 | 0.000 |  | 12.02 | 0.001 | 3 |
| pEnvGoaI | 0.708 | 0.956 | 225.09 | 0.000 |  | 0.32 | 0.572 | 0 |
| pEnvGoAi | 0.653 | 0.962 | 376.06 | 0.000 |  | 10.96 | 0.001 | 7 |
| pEnvGoAI | 0.701 | 0.954 | 228.13 | 0.000 |  | 0 | 0.950 | 1 |
| pEnvGOai | 0.676 | 0.958 | 351.2 | 0.000 |  | 3.28 | 0.070 | 11 |
| pEnvGOaI | 0.726 | 0.945 | 191.98 | 0.000 |  | 3.64 | 0.057 | 10 |
| pEnvGOAi | 0.68 | 0.952 | 317.86 | 0.000 |  | 2.33 | 0.127 | 18 |
| pEnvGOAI | 0.727 | 0.935 | 163.98 | 0.000 |  | 4.05 | 0.044 | 21 |
| pEnVgoai | 0.673 | 0.944 | 239.19 | 0.000 |  | 3.36 | 0.067 | 18 |
| pEnVgoaI | 0.721 | 0.939 | 153.81 | 0.000 |  | 2.12 | 0.145 | 7 |
| pEnVgoAi | 0.673 | 0.947 | 257.91 | 0.000 |  | 3.44 | 0.064 | 17 |
| pEnVgoAI | 0.714 | 0.94 | 163.52 | 0.000 |  | 0.91 | 0.340 | 13 |
| pEnVgOai | 0.719 | 0.953 | 226.52 | 0.000 |  | 1.96 | 0.162 | 3 |
| pEnVgOaI | 0.759 | 0.94 | 131.19 | 0.000 |  | 20.07 | 0.000 | 2 |
| pEnVgOAi | 0.712 | 0.948 | 216.85 | 0.000 |  | 0.77 | 0.381 | 10 |
| pEnVgOAI | 0.752 | 0.935 | 127.39 | 0.000 |  | 15.2 | 0.000 | 9 |
| pEnVGoai | 0.709 | 0.951 | 208.43 | 0.000 |  | 0.37 | 0.544 | 1 |
| pEnVGoaI | 0.744 | 0.943 | 135.35 | 0.000 |  | 9.57 | 0.002 | 1 |
| pEnVGoAi | 0.704 | 0.949 | 208.93 | 0.000 |  | 0.07 | 0.792 | 4 |
| pEnVGoAI | 0.739 | 0.94 | 134.56 | 0.000 |  | 7.25 | 0.007 | 2 |
| pEnVGOai | 0.73 | 0.941 | 177.71 | 0.000 |  | 5.17 | 0.023 | 9 |
| pEnVGOaI | 0.766 | 0.926 | 101.36 | 0.000 |  | 27.52 | 0.000 | 10 |
| pEnVGOAi | 0.723 | 0.931 | 157 | 0.000 |  | 2.93 | 0.087 | 29 |
| pEnVGOAI | 0.763 | 0.904 | 68.19 | 0.000 |  | 24.19 | 0.000 | 36 |
| pENvgoai | 0.693 | 0.937 | 179.88 | 0.000 |  | 0.19 | 0.666 | 10 |
| pENvgoaI | 0.747 | 0.927 | 96.04 | 0.000 |  | 10.78 | 0.001 | 6 |
| pENvgoAi | 0.702 | 0.937 | 174.24 | 0.000 |  | 0.01 | 0.909 | 5 |
| pENvgoAI | 0.741 | 0.924 | 98.53 | 0.000 |  | 7.63 | 0.006 | 9 |
| pENvgOai | 0.741 | 0.95 | 171.43 | 0.000 |  | 8.97 | 0.003 | 2 |
| pENvgOaI | 0.782 | 0.93 | 83.86 | 0.000 |  | 39.78 | 0.000 | 1 |
| pENvgOAi | 0.741 | 0.941 | 149.68 | 0.000 |  | 9.28 | 0.002 | 6 |
| pENvgOAI | 0.778 | 0.923 | 78.39 | 0.000 |  | 34.51 | 0.000 | 3 |
| pENvGoai | 0.734 | 0.947 | 158.09 | 0.000 |  | 5.4 | 0.020 | 0 |
| pENvGoaI | 0.776 | 0.926 | 72.69 | 0.000 |  | 29.36 | 0.000 | 2 |
| pENvGoAi | 0.735 | 0.941 | 143.01 | 0.000 |  | 5.85 | 0.016 | 0 |
| pENvGoAI | 0.766 | 0.922 | 74.57 | 0.000 |  | 21.93 | 0.000 | 4 |
| pENvGOai | 0.755 | 0.935 | 125.03 | 0.000 |  | 16.74 | 0.000 | 4 |
| pENvGOaI | 0.793 | 0.905 | 45.08 | 0.000 |  | 55.59 | 0.000 | 6 |
| pENvGOAi | 0.76 | 0.924 | 100.84 | 0.000 |  | 21.58 | 0.000 | 8 |
| pENvGOAI | 0.788 | 0.89 | 34.67 | 0.000 |  | 49.55 | 0.000 | 26 |
| pENVgoai | 0.754 | 0.927 | 91.45 | 0.000 |  | 13.66 | 0.000 | 1 |
| pENVgoaI | 0.788 | 0.904 | 37.83 | 0.000 |  | 39.28 | 0.000 | 2 |
| pENVgoAi | 0.755 | 0.92 | 82.04 | 0.000 |  | 15.19 | 0.000 | 5 |
| pENVgoAI | 0.784 | 0.897 | 36.08 | 0.000 |  | 36.12 | 0.000 | 8 |
| pENVgOai | 0.792 | 0.932 | 78.31 | 0.000 |  | 51.83 | 0.000 | 0 |
| pENVgOaI | 0.817 | 0.904 | 27.47 | 0.000 |  | 93.11 | 0.000 | 3 |
| pENVgOAi | 0.785 | 0.922 | 70.49 | 0.000 |  | 44.08 | 0.000 | 4 |
| pENVgOAI | 0.815 | 0.891 | 21.07 | 0.000 |  | 90.53 | 0.000 | 6 |
| pENVGoai | 0.78 | 0.931 | 77.32 | 0.000 |  | 33.21 | 0.000 | 0 |
| pENVGoaI | 0.812 | 0.895 | 20.95 | 0.000 |  | 73.23 | 0.000 | 1 |
| pENVGoAi | 0.778 | 0.922 | 66.67 | 0.000 |  | 32.05 | 0.000 | 0 |
| pENVGoAI | 0.806 | 0.887 | 19.14 | 0.000 |  | 65.64 | 0.000 | 3 |
| pENVGOai | 0.798 | 0.91 | 46.45 | 0.000 |  | 61.25 | 0.000 | 3 |
| pENVGOaI | 0.828 | 0.846 | 1.02 | 0.312 |  | 125.42 | 0.000 | 21 |
| pENVGOAi | 0.798 | 0.896 | 34.33 | 0.000 |  | 66.01 | 0.000 | 5 |
| pENVGOAI | 0.825 | 0.816 | 0.26 | 0.612 |  | 124.66 | 0.000 | 58 |
| Penvgoai | 0.582 | 0.934 | 289.21 | 0.000 |  | 50.73 | 0.000 | 75 |
| PenvgoaI | 0.704 | 0.945 | 169.31 | 0.000 |  | 0.05 | 0.816 | 7 |
| PenvgoAi | 0.653 | 0.955 | 287.46 | 0.000 |  | 9.02 | 0.003 | 18 |
| PenvgoAI | 0.705 | 0.95 | 177.33 | 0.000 |  | 0.11 | 0.740 | 4 |
| PenvgOai | 0.669 | 0.956 | 273.18 | 0.000 |  | 4.18 | 0.041 | 15 |
| PenvgOaI | 0.74 | 0.946 | 139.52 | 0.000 |  | 7.48 | 0.006 | 4 |
| PenvgOAi | 0.697 | 0.959 | 249.53 | 0.000 |  | 0.04 | 0.833 | 4 |
| PenvgOAI | 0.743 | 0.951 | 149.29 | 0.000 |  | 8.31 | 0.004 | 3 |
| PenvGoai | 0.665 | 0.958 | 264.06 | 0.000 |  | 4.87 | 0.027 | 1 |
| PenvGoaI | 0.722 | 0.956 | 165.71 | 0.000 |  | 1.93 | 0.164 | 1 |
| PenvGoAi | 0.684 | 0.959 | 243.39 | 0.000 |  | 0.98 | 0.321 | 0 |
| PenvGoAI | 0.717 | 0.957 | 170.18 | 0.000 |  | 1.05 | 0.305 | 1 |
| PenvGOai | 0.69 | 0.948 | 207.45 | 0.000 |  | 0.43 | 0.511 | 10 |
| PenvGOaI | 0.747 | 0.938 | 119.38 | 0.000 |  | 10.35 | 0.001 | 3 |
| PenvGOAi | 0.717 | 0.951 | 195.24 | 0.000 |  | 1.39 | 0.239 | 3 |
| PenvGOAI | 0.745 | 0.94 | 120.22 | 0.000 |  | 9.05 | 0.003 | 8 |
| PenVgoai | 0.692 | 0.937 | 162.52 | 0.000 |  | 0.23 | 0.632 | 13 |
| PenVgoaI | 0.747 | 0.93 | 91.1 | 0.000 |  | 9 | 0.003 | 3 |
| PenVgoAi | 0.714 | 0.94 | 146.73 | 0.000 |  | 0.77 | 0.382 | 4 |
| PenVgoAI | 0.75 | 0.925 | 78.65 | 0.000 |  | 10.39 | 0.001 | 5 |
| PenVgOai | 0.729 | 0.95 | 164.84 | 0.000 |  | 3.66 | 0.056 | 2 |
| PenVgOaI | 0.77 | 0.935 | 88.76 | 0.000 |  | 23.07 | 0.000 | 2 |
| PenVgOAi | 0.743 | 0.947 | 141.18 | 0.000 |  | 8.89 | 0.003 | 0 |
| PenVgOAI | 0.771 | 0.933 | 81.26 | 0.000 |  | 23.95 | 0.000 | 2 |
| PenVGoai | 0.719 | 0.945 | 146.47 | 0.000 |  | 1.42 | 0.234 | 1 |
| PenVGoaI | 0.753 | 0.94 | 98.91 | 0.000 |  | 11.05 | 0.001 | 0 |
| PenVGoAi | 0.727 | 0.946 | 140.39 | 0.000 |  | 2.9 | 0.089 | 0 |
| PenVGoAI | 0.75 | 0.934 | 89.56 | 0.000 |  | 9.89 | 0.002 | 0 |
| PenVGOai | 0.748 | 0.936 | 114.31 | 0.000 |  | 10.77 | 0.001 | 6 |
| PenVGOaI | 0.779 | 0.919 | 61.94 | 0.000 |  | 31.59 | 0.000 | 7 |
| PenVGOAi | 0.757 | 0.938 | 114.93 | 0.000 |  | 16.53 | 0.000 | 4 |
| PenVGOAI | 0.774 | 0.909 | 50.19 | 0.000 |  | 26.42 | 0.000 | 9 |
| PeNvgoai | 0.74 | 0.911 | 69.92 | 0.000 |  | 6.68 | 0.010 | 6 |
| PeNvgoaI | 0.789 | 0.879 | 17.53 | 0.000 |  | 37.87 | 0.000 | 14 |
| PeNvgoAi | 0.746 | 0.929 | 93.23 | 0.000 |  | 8.8 | 0.003 | 1 |
| PeNvgoAI | 0.775 | 0.9 | 38.4 | 0.000 |  | 24.9 | 0.000 | 12 |
| PeNvgOai | 0.769 | 0.932 | 86.06 | 0.000 |  | 22.85 | 0.000 | 1 |
| PeNvgOaI | 0.807 | 0.894 | 20.91 | 0.000 |  | 63.1 | 0.000 | 4 |
| PeNvgOAi | 0.775 | 0.932 | 78.4 | 0.000 |  | 26.47 | 0.000 | 2 |
| PeNvgOAI | 0.801 | 0.912 | 37.92 | 0.000 |  | 52.6 | 0.000 | 3 |
| PeNvGoai | 0.763 | 0.935 | 87.12 | 0.000 |  | 16.44 | 0.000 | 0 |
| PeNvGoaI | 0.798 | 0.901 | 26.91 | 0.000 |  | 45.41 | 0.000 | 2 |
| PeNvGoAi | 0.764 | 0.932 | 77.76 | 0.000 |  | 16.87 | 0.000 | 1 |
| PeNvGoAI | 0.788 | 0.914 | 43.87 | 0.000 |  | 33.71 | 0.000 | 1 |
| PeNvGOai | 0.782 | 0.918 | 56.17 | 0.000 |  | 33.14 | 0.000 | 1 |
| PeNvGOaI | 0.816 | 0.86 | 4.72 | 0.030 |  | 78.38 | 0.000 | 9 |
| PeNvGOAi | 0.787 | 0.922 | 58.51 | 0.000 |  | 39.12 | 0.000 | 2 |
| PeNvGOAI | 0.808 | 0.879 | 13.81 | 0.000 |  | 63.83 | 0.000 | 13 |
| PeNVgoai | 0.787 | 0.9 | 33.07 | 0.000 |  | 35.44 | 0.000 | 3 |
| PeNVgoaI | 0.818 | 0.854 | 2.87 | 0.090 |  | 71.09 | 0.000 | 9 |
| PeNVgoAi | 0.793 | 0.906 | 34.77 | 0.000 |  | 41.55 | 0.000 | 0 |
| PeNVgoAI | 0.817 | 0.865 | 5.18 | 0.023 |  | 73.48 | 0.000 | 4 |
| PeNVgOai | 0.807 | 0.915 | 38.66 | 0.000 |  | 62.16 | 0.000 | 0 |
| PeNVgOaI | 0.836 | 0.859 | 1.44 | 0.231 |  | 118.52 | 0.000 | 4 |
| PeNVgOAi | 0.811 | 0.912 | 32.19 | 0.000 |  | 67.07 | 0.000 | 2 |
| PeNVgOAI | 0.83 | 0.874 | 5.21 | 0.023 |  | 98.53 | 0.000 | 7 |
| PeNVGoai | 0.793 | 0.916 | 41.54 | 0.000 |  | 37.91 | 0.000 | 0 |
| PeNVGoaI | 0.822 | 0.851 | 1.7 | 0.192 |  | 73.25 | 0.000 | 6 |
| PeNVGoAi | 0.795 | 0.911 | 35.88 | 0.000 |  | 40.98 | 0.000 | 0 |
| PeNVGoAI | 0.819 | 0.861 | 3.84 | 0.050 |  | 74.9 | 0.000 | 4 |
| PeNVGOai | 0.817 | 0.896 | 19.45 | 0.000 |  | 78.87 | 0.000 | 1 |
| PeNVGOaI | 0.85 | 0.787 | 8.94 | 0.003 |  | 171.5 | 0.000 | 28 |
| PeNVGOAi | 0.819 | 0.895 | 18.59 | 0.000 |  | 85.56 | 0.000 | 2 |
| PeNVGOAI | 0.844 | 0.8 | 4.49 | 0.034 |  | 147.7 | 0.000 | 32 |
| PEnvgoai | 0.578 | 0.951 | 420.7 | 0.000 |  | 61.75 | 0.000 | 52 |
| PEnvgoaI | 0.682 | 0.957 | 242.82 | 0.000 |  | 1.31 | 0.253 | 11 |
| PEnvgoAi | 0.611 | 0.96 | 422.14 | 0.000 |  | 34.04 | 0.000 | 29 |
| PEnvgoAI | 0.687 | 0.955 | 232.24 | 0.000 |  | 0.77 | 0.380 | 9 |
| PEnvgOai | 0.65 | 0.965 | 384.95 | 0.000 |  | 11.8 | 0.001 | 8 |
| PEnvgOaI | 0.724 | 0.952 | 182.08 | 0.000 |  | 2.88 | 0.090 | 4 |
| PEnvgOAi | 0.666 | 0.963 | 335.54 | 0.000 |  | 5.35 | 0.021 | 9 |
| PEnvgOAI | 0.726 | 0.954 | 189.01 | 0.000 |  | 3.33 | 0.068 | 2 |
| PEnvGoai | 0.654 | 0.966 | 354.09 | 0.000 |  | 9.41 | 0.002 | 1 |
| PEnvGoaI | 0.713 | 0.961 | 209.41 | 0.000 |  | 0.73 | 0.392 | 1 |
| PEnvGoAi | 0.663 | 0.967 | 347.39 | 0.000 |  | 6.11 | 0.014 | 2 |
| PEnvGoAI | 0.708 | 0.96 | 211.03 | 0.000 |  | 0.25 | 0.616 | 2 |
| PEnvGOai | 0.675 | 0.955 | 299.5 | 0.000 |  | 3.18 | 0.075 | 11 |
| PEnvGOaI | 0.73 | 0.946 | 161.39 | 0.000 |  | 4.49 | 0.034 | 4 |
| PEnvGOAi | 0.691 | 0.956 | 269.68 | 0.000 |  | 0.37 | 0.541 | 7 |
| PEnvGOAI | 0.741 | 0.939 | 138.39 | 0.000 |  | 8.7 | 0.003 | 11 |
| PEnVgoai | 0.68 | 0.947 | 218.41 | 0.000 |  | 1.62 | 0.203 | 4 |
| PEnVgoaI | 0.732 | 0.941 | 132.66 | 0.000 |  | 4.64 | 0.031 | 4 |
| PEnVgoAi | 0.684 | 0.948 | 213.26 | 0.000 |  | 1.08 | 0.298 | 18 |
| PEnVgoAI | 0.729 | 0.94 | 133.33 | 0.000 |  | 3.55 | 0.060 | 5 |
| PEnVgOai | 0.719 | 0.955 | 207.91 | 0.000 |  | 1.64 | 0.200 | 2 |
| PEnVgOaI | 0.759 | 0.941 | 116.1 | 0.000 |  | 17.33 | 0.000 | 2 |
| PEnVgOAi | 0.723 | 0.952 | 192.39 | 0.000 |  | 2.56 | 0.109 | 5 |
| PEnVgOAI | 0.759 | 0.938 | 109.7 | 0.000 |  | 16.62 | 0.000 | 6 |
| PEnVGoai | 0.709 | 0.957 | 210.35 | 0.000 |  | 0.36 | 0.550 | 1 |
| PEnVGoaI | 0.747 | 0.946 | 125.92 | 0.000 |  | 9.82 | 0.002 | 1 |
| PEnVGoAi | 0.712 | 0.958 | 210.16 | 0.000 |  | 0.59 | 0.441 | 1 |
| PEnVGoAI | 0.742 | 0.943 | 123.47 | 0.000 |  | 7.46 | 0.006 | 0 |
| PEnVGOai | 0.736 | 0.946 | 168.4 | 0.000 |  | 7.06 | 0.008 | 2 |
| PEnVGOaI | 0.768 | 0.927 | 84.79 | 0.000 |  | 24.35 | 0.000 | 6 |
| PEnVGOAi | 0.742 | 0.941 | 151.4 | 0.000 |  | 9.39 | 0.002 | 7 |
| PEnVGOAI | 0.774 | 0.905 | 53.1 | 0.000 |  | 31.01 | 0.000 | 17 |
| PENvgoai | 0.715 | 0.93 | 130.24 | 0.000 |  | 0.95 | 0.329 | 4 |
| PENvgoaI | 0.762 | 0.925 | 75.96 | 0.000 |  | 17.16 | 0.000 | 2 |
| PENvgoAi | 0.722 | 0.935 | 132.09 | 0.000 |  | 2.1 | 0.148 | 2 |
| PENvgoAI | 0.754 | 0.923 | 77.87 | 0.000 |  | 12.08 | 0.001 | 5 |
| PENvgOai | 0.748 | 0.944 | 132.29 | 0.000 |  | 10.8 | 0.001 | 0 |
| PENvgOaI | 0.792 | 0.918 | 50.53 | 0.000 |  | 44.94 | 0.000 | 0 |
| PENvgOAi | 0.752 | 0.939 | 117.62 | 0.000 |  | 12.73 | 0.000 | 1 |
| PENvgOAI | 0.786 | 0.92 | 57.19 | 0.000 |  | 36.73 | 0.000 | 3 |
| PENvGoai | 0.741 | 0.945 | 130.61 | 0.000 |  | 7.31 | 0.007 | 1 |
| PENvGoaI | 0.784 | 0.919 | 47.55 | 0.000 |  | 32.63 | 0.000 | 4 |
| PENvGoAi | 0.743 | 0.946 | 130.47 | 0.000 |  | 7.76 | 0.005 | 0 |
| PENvGoAI | 0.771 | 0.927 | 68.93 | 0.000 |  | 22.19 | 0.000 | 2 |
| PENvGOai | 0.761 | 0.931 | 96.28 | 0.000 |  | 19.07 | 0.000 | 0 |
| PENvGOaI | 0.799 | 0.891 | 24.17 | 0.000 |  | 55.64 | 0.000 | 7 |
| PENvGOAi | 0.768 | 0.931 | 94.88 | 0.000 |  | 23.78 | 0.000 | 2 |
| PENvGOAI | 0.798 | 0.885 | 21.43 | 0.000 |  | 55.08 | 0.000 | 17 |
| PENVgoai | 0.768 | 0.922 | 67.68 | 0.000 |  | 20.68 | 0.000 | 0 |
| PENVgoaI | 0.797 | 0.908 | 34.4 | 0.000 |  | 45.1 | 0.000 | 1 |
| PENVgoAi | 0.778 | 0.915 | 54.62 | 0.000 |  | 30.47 | 0.000 | 1 |
| PENVgoAI | 0.796 | 0.899 | 28.18 | 0.000 |  | 44.54 | 0.000 | 4 |
| PENVgOai | 0.791 | 0.931 | 68.78 | 0.000 |  | 44.71 | 0.000 | 0 |
| PENVgOaI | 0.818 | 0.898 | 19.81 | 0.000 |  | 81.99 | 0.000 | 3 |
| PENVgOAi | 0.797 | 0.919 | 50.29 | 0.000 |  | 53.31 | 0.000 | 2 |
| PENVgOAI | 0.816 | 0.893 | 18.07 | 0.000 |  | 74.76 | 0.000 | 3 |
| PENVGoai | 0.781 | 0.932 | 71.65 | 0.000 |  | 29.89 | 0.000 | 0 |
| PENVGoaI | 0.812 | 0.898 | 19.51 | 0.000 |  | 65.43 | 0.000 | 2 |
| PENVGoAi | 0.784 | 0.925 | 61.34 | 0.000 |  | 34.92 | 0.000 | 2 |
| PENVGoAI | 0.807 | 0.892 | 20.03 | 0.000 |  | 60.32 | 0.000 | 1 |
| PENVGOai | 0.801 | 0.916 | 46.47 | 0.000 |  | 59.62 | 0.000 | 0 |
| PENVGOaI | 0.833 | 0.846 | 0.42 | 0.515 |  | 123.23 | 0.000 | 9 |
| PENVGOAi | 0.808 | 0.904 | 31.73 | 0.000 |  | 75.84 | 0.000 | 4 |
| PENVGOAI | 0.825 | 0.81 | 0.51 | 0.473 |  | 105.3 | 0.000 | 40 |

PHQ-9: the Patient Health Questionnaire-9; ESQ-RSC: Eysenck personality Questionnaire-revised Short Scale for Chinese; PSSG: Questionnaires of psychosocial stress survey for groups.

*Statistical analysis methods：fs-QCA.
